# Guide to developing research recruitment strategies with the experts

**DOI:** 10.1136/bmjno-2025-001475

**Published:** 2026-03-05

**Authors:** Gita Ramdharry, Mie Rizig, Babikir Osman

**Affiliations:** 1 Department of Neuromuscular Disorders, UCL Queen Square Institute of Neurology, London, UK; 2 Queen Square Centre for Neuromuscular Diseases, National Hospital for Neurology and Neurosurgery, University College Hospitals NHS Trust, London, UK; 3 UCL Queen Square Institute of Neurology, London, UK; 4 Patient Representative, National Hospital for Neurology and Neurosurgery, University College Hospitals NHS Trust, London, UK

**Keywords:** NEUROMUSCULAR

## Abstract

**Background:**

The aim of this project was to work in partnership with people living with neurological diseases, from backgrounds that are racially minoritised in the UK, to co-produce a research recruitment strategy. Their experiences living with a neurological disease were vital to the co-production, plus their deep understanding of their community, culture and factors impacting engagement.

**Methods:**

Six patient partners agreed to participate in three workshops that aimed to explore experiences of research, barriers and community concerns, culminating in ideas for strategies and actions to address the challenges.

**Results:**

Workshop 1 identified key issues around knowledge, communication, trust, shame and personal choice. Discussion and consensus in workshops 2 and 3 resulted in eight strategy areas: (1) Access to specialist clinics, (2) Setting up Patient and Public Involvement groups for specific studies or programmes, (3) Access to information on research, (4) Accessible and attractive information, (5) Incentives for participation in research, (6) Involving family members in decisions on research, (7) Communicating research outcomes and (8) Cultural sensitivity and diversity in the research team.

**Conclusions:**

The process of co-production formulated a strategy to promote engagement and increase ethnic diversity within the context of neurological disease research. Modification and refinement for other areas and contexts is recommended, in partnership with people who live with the day-to-day experiences of specific diseases.

## Background

 There is evidence of poor representation of people from racially minoritised backgrounds in clinical research.^1 2^ People with complex disabilities or rarer diseases are often considered ‘hard to reach’ and the intersection of characteristics further compounds the issue.^3^ The people who can help to develop the best strategies for engagement are those with specific lived experience.^4 5^

Here, we present an example of a Patient, Public Involvement and Engagement (PPIE) activity with people attending our neuromuscular diseases (NMDs) service, from racialised minority backgrounds. Many NMDs are inherited, with racial and cultural factors influencing some rare genetic subtypes and/or access to specialist services. We recognise the need for optimising recruitment to research to include individuals from diverse communities and backgrounds, aiming for equitable access to specialist care and treatment in the longer term.^1^

We focused on awareness and engagement with research activities through a process of reflection on individual experiences, drawing on their deep understanding of their community and culture. These discussions were used to facilitate the development of ideas to formulate a strategy for involvement, engagement and inclusion in research.^6^

## Methods

Our engagement team includes someone living with NMD, who advised on the plan for the project. We invited people living with NMDs from racially minoritised backgrounds from our clinics to form the PPIE panel for this work. Individuals were approached purposively to ensure diversity of background and socioeconomic status.

Workshops were facilitated by the lead author, held online using Zoom conferencing software, and recorded and then transcribed. If a panel member was unable to attend the workshops, individual interviews were offered. Transcripts were coded to identify key experiences and issues. The focus of each workshop is detailed below:

### Workshop 1

Exchange of experiences and ideas

- Awareness of research
- Community concerns
- Barriers to engagement
- Early engagement ideas

Key issues from workshop 1 were collated with feedback and confirmation of accuracy checked by our coauthor with lived experience.

### Workshop 2

Bringing strategy ideas together with actions

- Presentation and review of the collated ideas from workshop 1
- Developing strategies from the key ideas
- Collating suggested actions under each strategy
- Dissemination of the draft strategy after workshop 2

Strategy ideas and actions were grouped and again, confirmation of accuracy was checked by our coauthor with lived experience. A presentation of the preliminary strategy was prepared for workshop 3.

### Workshop 3

Agreeing the final strategy and training

- Presentation of the preliminary strategy
- Debate, discussion and final agreement
- Discussion on format of a launch of the strategy
- Suggestions on training needs for patient engagement

Final strategy actions were collated into a document. This was sent to all panel members for amendment as required.

## Results

The advisory panel comprised six people who live with NMD who attended either group workshops or individual interviews. They came from a mix of socioeconomic, ethnic and cultural backgrounds: 2 South Asian, 1 East Asian, 1 Middle Eastern, 1 East African, 1 British Caribbean. The group was 50:50 male to female, two were in full-time employment, one in part-time employment, two were not employed receiving disability support payments, and one was not eligible for support payments due to visa status, so relying on family support.

### Workshop 1

Five areas were identified from the discussion that explored factors impacting engagement with research: Knowledge, Communication, Trust, Shame and Personal Choice. These discussion points are presented in online supplemental figure 1, with illustrative quotes.

#### Knowledge

Attending specialist clinics was key to gaining deeper understanding of their condition and having the opportunity to access research. For all of them, this was a gateway to engagement, allowing them access to specialist information which can be hard to find on their own. There were variations in understanding the purpose of research they were currently participating in. Online resources and social media groups were sought by most, with significant amounts of time spent searching for more information on their condition.

#### Communication

The panel identified a need for researchers to proactively reach out to communities to engage, but to not assume awareness that research takes place. This will vary between communities, and the panel suggested including extended family in decision-making for participation. They made recommendations on diversity in who and how research information is delivered, valuing the perspectives of people with similar conditions who have participated in research. Translation of study materials was discussed, but they raised the importance of interpreters during the decision-making/consent process.

#### Trust

The panel highlighted the importance of reassurance that clinical care will not be impacted by the decision outcome. There were diverse views on the issue of trust in medicine, and by extension, medical research. In some communities, there is absolute trust in health professionals which can facilitate research engagement. In others, there is deep mistrust based on experiences of historical mistreatment by colonial authorities plus ongoing discrimination. The panel reflected on the phenomenon of vaccine hesitancy during the COVID-19 pandemic, which was more prevalent in some minority communities in the UK.

#### Shame

A suggested barrier to engagement was the shame of having a disease, with a tendency to avoid highlighting it. Some extended communities perpetuate this, tending to conceal family with disease and disability. A panel member shared that they had not disclosed their condition to family members.

### Personal choice

Panel members highlighted that for some individuals, the practicalities of living with a disabling disease were prioritised over gaining condition knowledge or engaging in research.

### Workshops 2 and 3

Strategy ideas and actions were generated to address the issues identified. Initial debate and discussion took place in workshop 2 and were summarised in a prototype strategy document. The final document was presented, discussed and agreed in workshop 3 and emailed to all panel members for final amendments. The strategy and associated actions are summarised below with additional detail in table 1.

**Table 1:** Agreed research participant recruitment strategies to increase ethnic diversity, with suggested actions

Strategy	Actions
(1) Access to specialist clinics	Widening access through virtual appointments and telemedicine.
(2) Setting up patient and public involvement (PPI) groups for specific studies or programmes	Recruit members from diverse groups (ethnicity, educational level, etc).
	Provide information on expectations and what is required for people to consider before committing: processes, resources, purpose.
	Provide training on participation and roles.
	Cost for payments/vouchers to PPI members for the time and expenses for activities as part of the advisory work in any grant applications.
(3) Access to information on research	Open resources and ‘friendly’ information sites for health professionals and patients.
	Linking in with charities and other groups such as the Neurological Alliance and Rare Disease Alliance to disseminate information on research.
	Linking in with disease-specific online communities.
	Videos or leaflets with people from diverse backgrounds talking about their experience of being involved in research (actors can be used if participants are not comfortable appearing).
	Introduce potential participants to study websites or YouTube channels when inviting them to participate.
(4) Accessible and attractive information	Use of digital communication means but also complementing with paper-based resources to span range of digital literacy.
	Simpler language in research paperwork, and inclusion of a headline summary before the detailed information.
	Translation of research Information into other languages (including Braille, video subtitles and British Sign Language) or signposting to where different languages can be requested.
	Use of interpreter services in screening and consent processes so questions can be asked and answered, and the interpreter can check understanding.
(5) Incentives for participation in research	Transparency on the purpose, requirements of participating and right to withdraw.
	Payment of expenses (eg, transportation) and providing refreshments if it is a long data collection session.
	Provide vouchers or payment in recognition and respect for the person’s time.
	Highlighting the potential contribution to the individual, wider community and society.
	Welcoming and pleasant environment
(6) Involving family members in decisions on research	Producing information for family members, particularly elders, on why their relative is being approached to participate in research.
	A range of formats for information: digital, videos and paper-based, different languages, availability of interpreters.
	Reassurance around participation: payment of expenses, vouchers, burden of involvement, right to withdraw.
(7) Communicating research outcomes	Prioritise this in the study plan and milestone.
	Use charity communication methods and online peer groups.
	Webinars, online videos and social media posts.
	Consider engaging with media professionals and social media influencers.
(8) Cultural sensitivity and diversity in the research team	Sensitivity in how a diagnosis is discussed; ensure participant feels comfortable; awareness of issues such as shame in some communities.
	Avoid generalisation, judgement or preconceived notions; respect each individual’s experiences.
	Education programme for research delivery teams.
	Explore opportunities for diversity in the research team, particularly if approaching a specific community; avoid tokenism.

Access to specialist services was identified as a key gateway to research participation with suggestions around widening access through virtual appointments and telemedicine.

Setting up patient and public involvement groups for specific studies or programmes was recommended, ensuring diversity within groups. Information should be available on the expectations, reimbursement, training and support.

Access to information on research should be via open resources with accessible information on websites with diversity of individuals represented. The panel also recommended linking with charities to signpost to project websites and pages. Accessible and attractive information should be available via digital communication means, but they recognised that paper-based resources are still required to meet a range of digital literacy in potential participants. Headline summaries were recommended for study information documents, with options for translation and available interpreters during the consent process.

Incentives for participation in research should be considered in addition to the usual reimbursement for travel for research participants. They recommended vouchers or payment for participation to support people with low incomes, who will carry disproportionate financial and time burden for research visits. Transparency on the purpose and emphasising altruistic aspects of participation were also deemed important. Involving family members in decisions on research was something research teams should consider.

Communicating research outcomes should be prioritised in the study plan with wider delivery methods, for example, via charities, using a variety of online materials and engagement with digital media-savvy individuals.

Diversity and cultural sensitivity in the research team through education and focused recruitment of personnel should be considered, particularly if studies focus on specific communities.

## Discussion

Here, we present the output from a co-production exercise, presenting eight strategies with examples of actions to promote greater diversity in research cohorts. Flexible engagement methods were used to meet the needs and preferences of the panel members. Every effort was made to ensure an ethnic and cultural mix on the panel, but there cannot be assumptions that these voices speak for all backgrounds. We recommend research undertaken with specific communities build on this work to modify and refine a strategy with patient partners to ensure it fits with their specific values, beliefs and context. A key limitation became apparent during this project, in that five of the six panel members were highly educated, to at least degree level. We achieved a varied socioeconomic mix, but it is stark that this could be at odds with the educational background of some members. To optimise accessibility of information, engaging with people from a wider level of education is recommended for any further work building on this first strategy.

An important take-home message from this work is not just the strategy itself, but the process of partnership in co-production that can be extended to other conditions. Embedding partnership throughout the research cycle allows for adjustment and improvement of outcomes. Research programmes should plan and incorporate funding, time and resources to effectively implement these strategies.^7^ People living with intersecting experiences and realities, such as those described here, are best placed to advise us on how we can increase diversity of participation in research.

## Supplementary material

10.1136/bmjno-2025-001475 online supplemental file 1
