# Supplementary figures and images for: Guide to developing research recruitment strategies with the experts
Source: BMJ Neurol Open. 2026 Mar 5;8(1):e001475. doi: 10.1136/bmjno-2025-001475 (PMC12970071; doi:10.1136/bmjno-2025-001475)

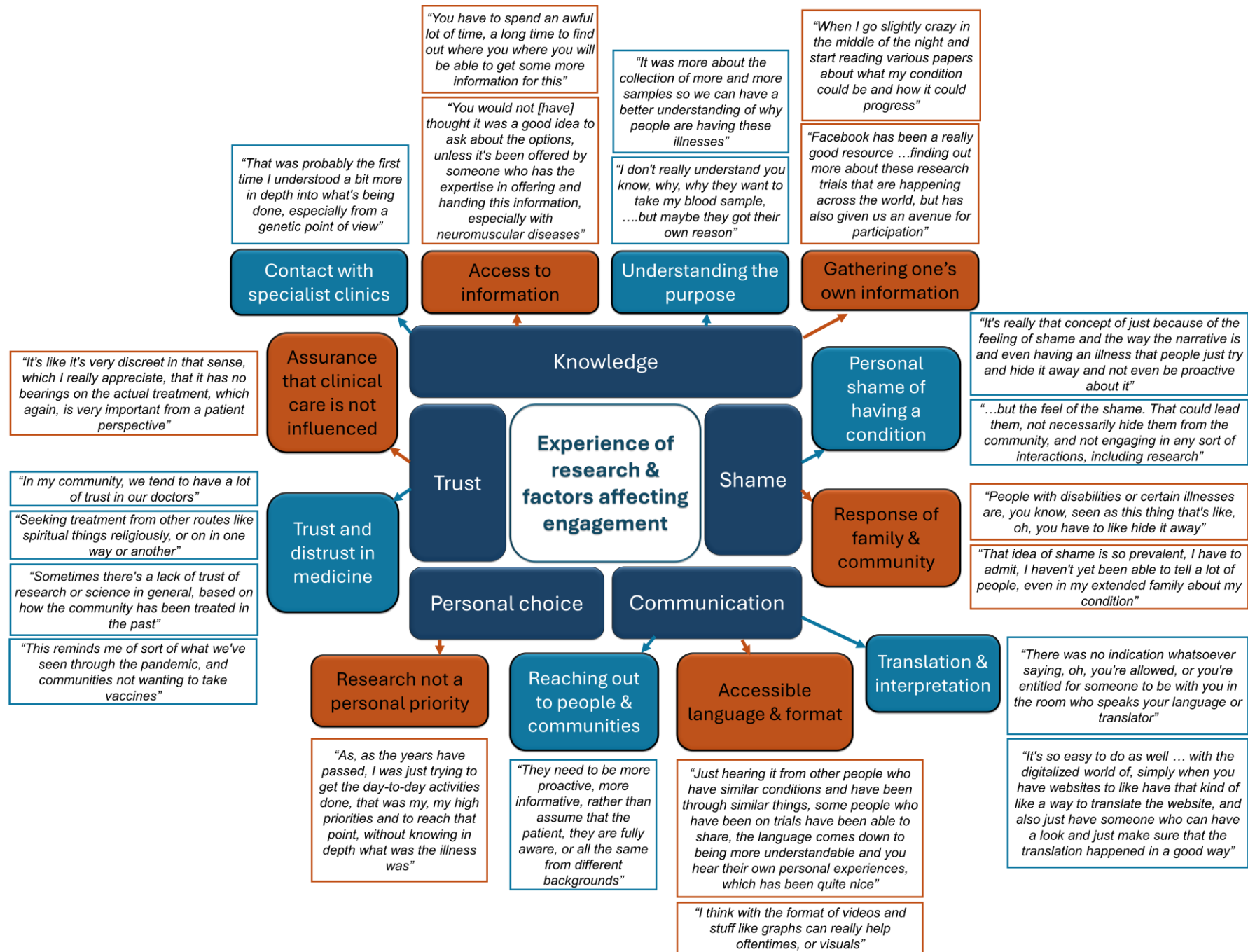

**Supplemental Figure 1:** Workshop 1 outputs, with discussion points and illustrative quotes

Supplement: online supplemental file 1 [file bmjno-8-1-s001.pdf]
